# Outcomes after stenting of renal artery stenosis in patients with high-risk clinical features

**DOI:** 10.1186/s43044-024-00435-z

**Published:** 2024-01-18

**Authors:** Calin Homorodean, Mihai Claudiu Ober, Mihail Spinu, Maria Olinic, Dan-Alexandru Tataru, Horea Laurentiu Onea, Alexandru Achim, Leontin Florin Lazar, Romana Homorodean, Balasz Deak, Dan Mircea Olinic

**Affiliations:** 1 Medical Clinic No. 1, “Iuliu Hatieganu” University of Medicine and Pharmacy, Cluj-Napoca, Cluj, Romania; 2 Cluj County Emergency Clinical Hospital, 3-5, Clinicilor Street, 400006 Cluj-Napoca, Cluj, Romania

**Keywords:** Renal artery disease, Renal artery stenting, Cardiac destabilization syndromes, Rapidly declining renal function

## Abstract

**Background:**

In patients with renal artery stenosis, revascularization was seen as a mean to improve outcomes, but large studies failed to show significant benefit in general population. However, data on benefits of renal artery stenting in patients with high-risk features, such as rapidly declining renal function and cardiac destabilization syndromes, are limited, as they were excluded from trials. In this descriptive study, we aimed to evaluate short- and long-term outcomes in high-risk patients with renal artery stenosis, treated by angioplasty and stenting. We have retrospectively interrogated our local databases for renal artery percutaneous interventions; patients at high-risk (rapidly declining renal function; stable chronic renal failure and bilateral renal artery disease; severe hypertensive crisis) were selected for the current analysis.

**Results:**

Of 30 patients undergoing renal artery stenting, 18 patients were deemed "high-risk." On short term, good in-hospital control of hypertension and cardiac stabilization were obtained in all patients. Renal function improved significantly only in patients admitted with rapidly declining renal function, with significant creatinine level fall from median 3.98 mg/dL to 2.02 mg/dL, *p* = 0.023. However, for the whole group, creatinine change was non-significant (− 0.12 mg/dL, *p* = NS). On the long term, five patients (27.8%) ended-up on chronic hemodialysis and six patients died (33.3%) after a median of 20 months. No death occurred during the first year after the procedure.

**Conclusions:**

Percutaneous procedures are feasible and safe in patients with high-risk renal artery stenosis, especially in those with rapidly declining renal function, probably saving some of them from the immediate need for renal replacement therapy, but long-term results are negatively influenced by the precarious general and cardio-vascular status of these patients and by the pre-existing significant renal parenchymal disease, non-related to the renal artery stenosis.

## Background

Renal artery stenosis (RAS) is a frequent pathology, found in post-mortem studies in 27% of patients older than 50 years [1] or incidentally discovered during an aortography [2], usually of atherosclerotic origin.

RAS may be clinically silent or may result in more or less severe arterial hypertension. Ischemic nephropathy with renal failure emerges when RAS affects the entire functional renal mass (bilateral RAS or single functional kidney) [2]. Acute decompensation is present in 23% of the patients with RAS, leading to rapidly declining renal function [3] or systemic effects (cardiac destabilization syndromes—flash pulmonary edema (FPE), aortic syndromes, acute coronary syndromes, or recurrent congestive heart failure) [4].

Clinical manifestations of RAS can be addressed either by medical treatment or by revascularization. The aims of RAS revascularization are hypertension control, prevention of cardiac destabilization syndromes, and preservation of renal function, with the ultimate goal of reducing cardiovascular events and mortality.

Randomized trials show little long-term benefit of revascularization over medical therapy alone in general RAS population [5, 6]. However, patients included in the randomized trials are clinically less severe, because they should satisfy the perceived equipoise of eligibility for medical treatment or stenting. Consequently, high-risk patients are usually excluded from randomized trials [6, 7].

Data from observational studies suggest that high-risk clinical conditions associated with RAS most likely benefit from revascularization [1], but even in these high-risk patients, some clinical patterns will benefit more than others [8]. Hence, there is a need for further exploration of the ideally suitable patient for renal revascularization.

The study is a descriptive analysis of short- and long-term outcomes of angioplasty in RAS patients with high-risk features.

## Methods

We have retrospectively interrogated our hospital and interventions databases for renal artery percutaneous interventions performed on stenosis above 70% on angiography, between January 2010 and September 2022.

The patients at the highest risk were selected for the current analysis. High-risk criteria were considered as follows:

1. *Rapidly declining renal function*, when serum creatinine increased more than 0,5 mg/dL, referred to a baseline reading, within the previous 6 months, outside an acute decompensation state.
2. *Stable chronic renal failure AND RAS affecting the entire functional renal mass,* meaning significant (> 70% at angiography), bilateral renal artery disease or on unique functional kidney and estimated glomerular filtration rate (eGFR) of less than 60 ml/min.
3. *Severe hypertensive crisis* was considered in patients presenting with high blood pressure levels inducing cardiac destabilization syndromes (acute coronary syndromes or acute heart failure, more often acute pulmonary edema) or hypertensive encephalopathy, with need for intravenous drugs for blood pressure control. Patients should have had at least one hospital admission for the above syndromes, with no obvious explanations such as non-adherence, left ventricular ejection fraction < 40%, or valvular heart disease.

Of these patients, a *very high-risk category* was selected, undergoing revascularization on a unique kidney or with residual contralateral occlusion (functionally unique kidney).

For the high-risk patients, demographic, clinical, and laboratory data were obtained from the hospital database. The presence of *diabetes mellitus* and *other arterial territory involvement* (coronary, peripheral, or cerebral artery disease) were assessed as an insight into the etiology of both renal failure and renal artery disease. In addition, the completeness of renal revascularization was checked, defined as a non-significant residual lesion on both renal arteries at the end of the procedure(s); unique kidney revascularization was deemed incomplete.

The occurrence of the following adverse events was recorded during hospitalization: death, myocardial infarction, contrast-induced nephropathy (CIN) or need for renal replacement therapy. At follow-up, need for renal replacement therapy and death of any cause were the reported outcomes.

In addition, serum creatinine evolution during the index hospitalization was analyzed by reporting four values: baseline level (recorded during the previous six months, obtained from patient’s medical records), preprocedural (a value within 24 h before stenting), postprocedural (the highest level after the stenting procedure), and the last pre-discharge value, when the patient was considered stable.

As the rapidly declining renal function was perceived as a particular condition which might derive higher benefit from revascularization, the evolution of creatinine level during hospitalization was stratified by presence or absence of this condition as reported to the baseline value.

Any variation of serum creatinine of more than 0.5 mg/dL, between any time points, was considered significant. This cut-off was used for the definition *of initial rapidly declining renal function*, for the *definition of CIN* at the highest point of post-procedure creatinine level, and for the *pre-discharge creatinine level* related to the baseline or pre-procedure values. The actual time between the creatinine check-points varied between patients. Creatinine levels of 1.5 mg/dL or less were considered normal or near normal at all time points.

The vital status of the patients as on September 2022 was obtained from the National Health Insurance electronic records. The living patients were contacted by phone, and their current health status was assessed, focusing on hypertension control, current medication and the need for renal replacement therapy. For the dead patients, time to death was estimated from medical records and/or family contact. Death within one year after the index procedure was taken into consideration, irrespective of the presumable cause, in order to avoid large differences in follow-up duration between patients and to reduce the impact of other causes on mortality. Follow-up length was calculated in months between the index procedure and the time of the study or estimated time of death for the living and dead patients, respectively.

Interventional procedures were performed by interventional cardiologists, on Siemens Artis Zee angiograph (Siemens Healthineers, Erlangen, Germany).

### Statistical analysis

Continuous data were expressed as median (inter-quartile range, Q1—Q3), while categorical data were expressed as number (percent of the respective group). Fisher's exact test was used to check the association between presumed risk factors (diabetes, baseline renal failure, a combination of both of the previous, initial rapidly declining renal function, and very high-risk status) and persistent/aggravated renal failure versus baseline or pre-procedure or renal replacement therapy at follow-up, as categorical outcome variables, by stratified univariate analysis. Binary logistic regression was used for univariate analysis of the risk factors for CIN. A p value less than 0.05 was considered statistically significant. Nonparametric, two-tailed, Mann–Whitney test for two independent samples was used to compare continuous variables (e.g., creatinine levels, blood pressure).

Statistical analysis was performed in SPSS 29.0 (IBM Corporation, Armonk, New York, USA).

The study complies with the Declaration of Helsinki on human research.

## Results

### Patients

During the study time interval of nearly 12 years, there were 32 renal artery interventions in 30 patients. Of these, 18 patients were deemed "high-risk," undergoing 19 procedures (one staged bilateral revascularization in the same patient), 11 were female (61.1%) and seven were male (38.9%), with a median age of 69.5 (64–74.5) years. Significant kidney disease (baseline serum creatinine above 1.5 mg/dL) before current high-risk status (baseline renal failure) was present in 10 patients (55.6%), Table 1.

**Table 1:** Baseline and procedural characteristics of the 18 high-risk patients

Median age (IQR)	69.5 (64–74.5) years
Gender	11 female (61.1%)
	7 male (38.9%)
Baseline significant kidney disease (serum creatinine > 1.5 mg/dL)	10 patients (55.6%)
High-risk criteria	Rapidly declining renal function—10 patients (55.6%)
	Chronic kidney disease + bilateral renal artery disease—4 patients (22.2%)
	Severe hypertensive crisis—4 patients (22.2%)
Very high-risk category (functionally unique kidney)	9 patients (50%)
Diabetes mellitus	6 patients (30.0%)
Preprocedural blood pressure (mmHg)	Systolic: 160 (156–180)
	Diastolic: 88 (80–100)
Renal artery stenosis severity (on angiography)	70–90%—10 patients (55.6%)
	90–99%—7 patients (38.9%)
	Occlusion—1 patient (5.6%)
Vascular access site	Right brachial—7 patients (38.9%)
	Femoral—6 patients (33.3%)
	Left radial—4 patients (22.2%)
	Right radial—in 1 patient (5.6%)
Complete revascularization	8 patients (44.4%)

The high-risk criteria were *rapidly declining renal function* in 10 patients (55.6%), *chronic kidney disease with bilateral renal artery disease* in 4 patients (22.2%), and *severe hypertensive crisis* in 4 patients (22.2%), Table 1. The *very high-risk category*, with virtually unique kidney revascularization, included nine patients (50%), Table 1. No association was found between very high-risk status and acute worsening of renal failure (*p* = 1).

In all patients, the lesions were most probably atherosclerotic. Most of the patients had other vascular territory involvement (14 patients, 77.8%), suggesting a high general atherosclerotic burden. Six patients had diabetes mellitus (30.0%), Table 1.

### Stenting procedure

Concerning procedural aspects, the most frequent approach was right brachial in 7 patients (38.9%), followed by femoral in 6 patients (33.3%), left radial in 4 patients (22.2%), and right radial in 1 patient (5.6%), Table 1. Complete revascularization was obtained in 8 patients (44.4%), *i.e.,* all patients without contralateral occlusion, except one, in which the contralateral kidney was deemed irreversibly damaged, despite a non-occlusive lesion. In another patient, complete revascularization was achieved by staged procedures at 10-month intervals; in this patient, follow-up time was counted from the first procedure. Only one total occlusion was approached, unilateral disease, in a patient with severe previous renal failure (baseline creatinine 5.26 mg/dL), without significant improvement of the renal function, ending up on renal replacement therapy. There was one vascular access complication, one retroperitoneal hematoma that was conservatively managed.

### Renal function evolution

**Fig. 1:**
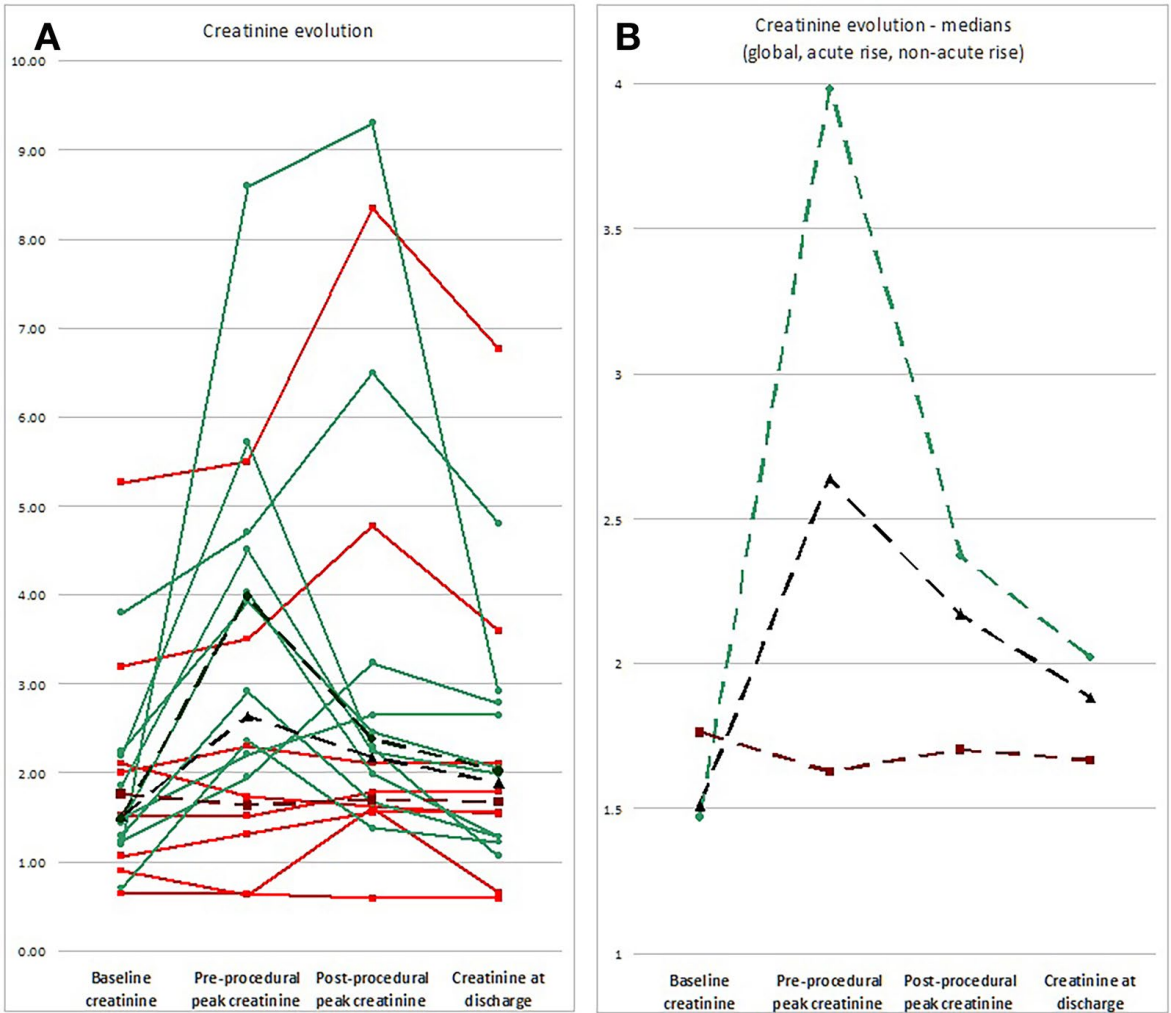
Creatinine level evolution of the 18 studied patients at the four time-points considered (baseline, preprocedural level, postprocedural maximum level, and at discharge). **A** Creatinine level for every patient, represented differently by the presence of the initial rapidly declining renal function: present (green, round dots), absent (red, square dots), and respective medians (corresponding dark colors, dotted line; black, dotted line—median for all patients). **B** The same representation for the medians only, rescaled for improved visibility (green, round dots—initial rapid decline present; dark red, square dots—initial rapid decline absent; black—whole group)

Despite a slightly lower median baseline creatinine level (1.47 mg/dL vs. 1.76 mg/dL) in patients showing *rapidly declining renal function*, preprocedural creatinine was significantly higher in these patients (3.98 mg/dL vs. 1.63 mg/dL, *p* = 0.021, Fig. 1), reflecting the abrupt renal function decline (Table 2).

**Table 2:** Creatinine level evolution, divided by the presence or absence of rapidly declining renal function before the procedure—values are expressed as median (inter-quartile range) for the respective population

	Rapidly declining renal function N = 10	Stable renal function N = 8	*p *value
Baseline creatinine (mg/dL)	1.47 (1.25–2.12)	1.76 (1.03–2.38)	NS
Pre-procedure creatinine (mg/dL)	3.98 (2.50–4.65)	1.63 (1.14–2.60)	0.02
Postprocedural peak creatinine (mg/dL)	2.37 (2.05–3.08)	1.70 (1.60–2.77)	NS
Delta (peak—pre-procedure, mg/dL)	− 0.12 (− 1.90–0.64)	0.25 (− 0.07–1.05)	NS
Creatinine at discharge	2.02 (1.29–2.75)	1.67 (1.32–2.48)	NS
Delta (discharge—pre-procedure, mg/dL)	− 1.75 (− 2.68 to − 0.21)	0.06 (− 0.09–0.25)	0.05
Delta (discharge—baseline, mg/dL)	0.32 (− 0.12–1.11)	0.18 (− 0.10–0.42)	NS

Postprocedural rises of creatinine levels reaching CIN criteria occurred in six patients (33.3%), with a median rise of 1.28 (1.05–1.67) mg/dL. Of these, three patients had non-significant baseline renal failure (creatinine < 1.5 mg/dL), while only one had a normal creatinine level immediately before the procedure. Diabetes mellitus (a known risk factor for CIN) was present in four of these patients. Only one CIN patient was without diabetes *and* without pre-procedure renal failure. Of the tested risk factors, only diabetes was significantly associated with CIN in our patients (OR 10; CI 1.026–97.5; *p* = 0.048, Table 3). None of the patients required hemodialysis during the index hospitalization. Of the six CIN patients, in two, creatinine level remained significantly raised versus pre-procedure; in two, it returned to preprocedural level (but with persistent renal failure), while in the other two, a significant decrease before discharge versus initial value was noted.

**Table 3:** Univariate logistic analysis for the prediction of in-hospital CIN

	Odds ratio (OR)	Confidence intervals (CI)	*p* value
Diabetes Mellitus (Y/N)	10	1.026–97.5	0.048
Preprocedural creatinine (mg/dl)	1.4	0.831–2.463	0.196
Age (y)	0.99	0.886–1.107	0.990
Preprocedural Systolic BP (mmHg)	1.018	0.976–1.063	0.407

The patients were discharged after a median of 4 days (IQR 3–6 days, range 2–20 days), when deemed stabilized. Pre-discharge level of creatinine was slightly lower than that of pre-procedure, median difference − 0.12 (− 1.81–0.20) mg/dL (*p* = NS), with nine patients showing aggravated or steady significant renal failure (50%), while the other 9 presenting amelioration or non-significant renal failure. In two patients (11.1%), net aggravation of creatinine level was noted pre-discharge. However, in patients showing rapidly declining renal function, a significant fall of creatinine level was noted after the procedure (from median 3.98 mg/dL to 2.02 mg/dL, *p* = 0.023, Fig. 1), with a median difference between pre-discharge and pre-procedure creatinine of − 1.75 mg/dL, while it remained steady in the initially stable patients (delta = 0.06 mg/dL, *p* = 0.050 between subgroups, Table 2). Although, not all patients with rapidly declining renal function showed this significant drop after the procedure; it seems that the higher the preprocedural creatinine peak, the larger postprocedural fall (Fig. 1).

When baseline creatinine was taken as a reference, pre-discharge status seemed slightly worse, with a median creatinine rise of 0.19 (− 0.12–0.88) mg/dL (*p* = NS), 12 patients showing aggravated or steady kidney failure (66.7%), of which six patients (33.3%) presented net aggravation.

### Clinical events

On short-term, in hospital, no major clinical events occurred (death, myocardial infarction or need for renal replacement therapy). The patients with acute clinical picture (cardiac or renal) stabilized progressively, without need for other interventions or for intensive care admission, and were discharged at home.

During the long-term follow-up, six patients died (33.3%) after a median of 20 (15.2–23.7) months. However, none of them died during the first year after the procedure, suggesting that their death was not related to the index procedure or renal disease but to advanced age or severe comorbidities. Patients alive at the date of the study had a median follow-up of 20 (11.2–40.0) months.

Five patients (27.8%) ended-up on *chronic hemodialysis*, two of them with the worst baseline creatinine level, without significant amelioration after the procedure, (of which one died one year after the procedure), while the other three had an initial favorable course, with late acute deterioration after one month, 20 months and 32 months, respectively. In the latter patient, there was a documented acute occlusion of the stent, on a unique kidney, with failed recurrent percutaneous procedure. The only significant predictor of need for renal replacement therapy was the combination of diabetes and previous renal failure (OR = 18; *p* = 0.037), which suggests that this is related to the progression of the underlying renal parenchymal disease rather than to the renal vascular disease.

### Blood pressure control

Long-term control of hypertension was relatively good in all living patients contacted, with median systolic BP of 140 mmHg (IQR 125–150) and diastolic BP: 80 mmHg (IQR 72–90). Of note, one patient needed no hypertension medication, while ten others tolerate a renin-angiotensin blocking drug (for most of them, an angiotensin receptor blocker), which is important both for hypertension control and for the long-term prognostic effect of these drugs. Angiotensin Receptor Blockers or ACE inhibitors were significantly more frequently used in blood pressure control after stenting, while diuretics were significantly less used (Table 4).

**Table 4:** Blood pressure evolution and antihypertensive classes used before and after renal artery revascularization

	Pre-procedure	Follow-up contact	p-value
Blood pressure			
Systolic BP (mmHg)	160 (156–180)	140 (125–150)	0.007
Diastolic BP (mmHg)	88 (80–100)	80 (72–90)	0.078
Number of classes of drugs	4 (4–4.75)	4 (2.75–4)	0.138

Classes of drugs	n = 12*	n = 12	
ACEi/ARB	3 (25.0%)	10 (83.3%)	0.0112
Calcium channel blockers	10 (83.3%)	9 (75.0%)	0.78
Beta Blockers	10 (83.3%)	10 (83.3%)	0.84
Diuretics	11 (91.7%)	3 (25.0%)	0.002
Centrally-acting agents	10 (83.3%)	5 (41.7%)	0.068

*Comparison of the drug classes use for the 12 patients alive at follow-up

## Discussion

Our study showed that in patients with RAS and clinically high-risk features, renal artery stenting resulted in a very good short-term survival, without recurrence of cardiac destabilization syndromes.

Since most of the patients included in our analysis presented an acute or subacute clinical picture, the short-term effect of revascularization was evident during the hospital stay, as cardiac destabilization syndromes resolved and creatinine level stabilized. No major cardiovascular events (death or myocardial infarction) occurred. Stenting procedure was safe, with only one periprocedural vascular access complication, managed medically.

Nevertheless, long-term mortality was high, around 33% at two years of follow-up, suggesting the severe general cardiovascular disease burden of these patients. However, the high long-term mortality was consistent with other previous studies; Ritchi et al. showed a mortality of 56% in the medical group and 52% in the stenting group, at 3.8 years of follow-up [3].

Regarding the impact of stenting on renal function, some improvement was observed with respect to preprocedural creatinine levels. Yet, when compared to baseline levels (renal function before the rapid decline that had precipitated the hospitalization), pre-discharge median creatinine levels were somewhat higher. It seems that the initial acute rise of creatinine level is not fully reversible in all patients, with a few having their status aggravated by the procedure itself. However, significant creatinine level fall was noted in patients with initial rapidly declining renal function, suggesting the curative nature of the procedure in these patients. In the absence of revascularization, evolution would probably have been very fast toward end-stage renal disease (ESRD) in this subgroup, as suggested by the evolution of the patient with late stent occlusion.

Data from both randomized and observational trials underlie the need for appropriate selection of patients undergoing renal artery revascularization [6]. Randomized trials [9–12] have shown that revascularization does not alter clinical outcomes, compared to medical therapy alone, especially because the low-risk patients have little benefit [6, 9].

Nevertheless, observational studies provide consistent evidence of benefit of revascularization concerning different outcomes (major adverse events, ESRD, recurrence of cardiac destabilization syndromes) in RAS patients with high-risk features [3, 8, 13]. The discrepancy between randomized and observational trials is probably driven by the difference between patients included [4, 14].

In a large group of patients with RAS treated medically, Ritchie et al. showed that of the three high-risk presentations (FPE, rapidly declining renal function, and refractory hypertension), only FPE is a significant adverse prognostic marker [3]. We did not use refractory hypertension as high-risk criterion because it has various definitions and the data on its appropriateness as a high-risk feature are contradictory.

**Fig. 2:**
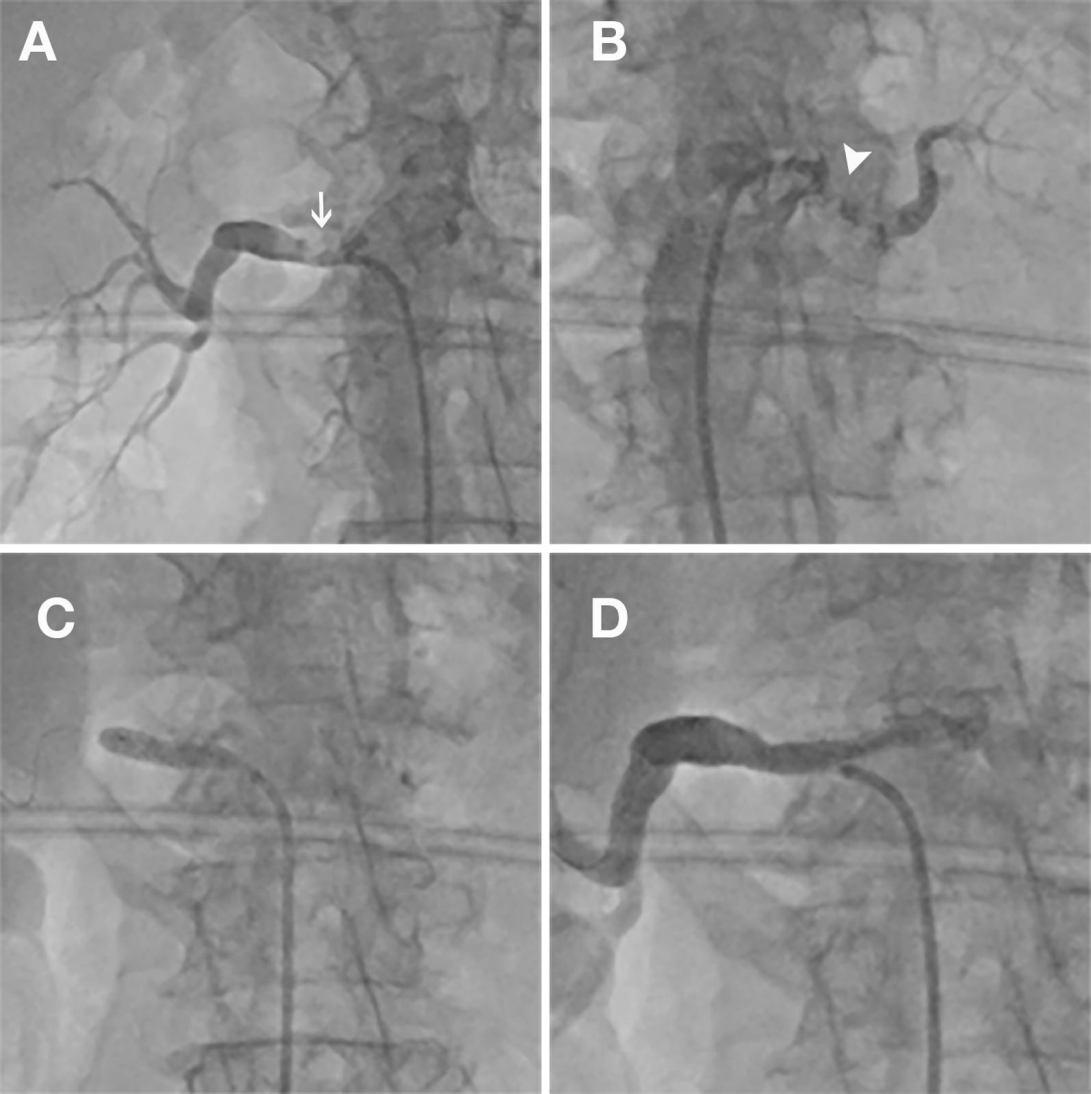
Bilateral renal artery stenosis in a patient with flash pulmonary edema and rapidly declining renal function. The tight stenosis on the right renal artery **(**panel **A**) was stented (panel** C**) with a good final result (panel** D**). The subocluded left renal artery (Panel** B**) subtended a small kidney (presumably, non-viable). Serum creatinine normalized 72 h after the procedure, there were no recurrences of pulmonary edema at 2 years of follow-up and hypertension was controlled with a single drug

An important concept for a good selection of patients most likely to benefit after revascularization is "global renal ischemia" [1]. In unilateral RAS, the hypertension induced by angiotensin-aldosterone activation is partially compensated by pressure natriuresis in the contralateral, non-ischemic kidney [15]. This is not the case in severe bilateral RAS (Fig. 2). Vassallo et al. [8] underlined the importance of severe bilateral RAS in predicting a revascularization benefit, showing that, even in high-risk patients, likelihood of benefit is higher in patients with severe bilateral RAS and low levels of proteinuria. Therefore, we also included in our research patients with chronic renal failure *and* significant, bilateral renal artery disease (or unique functional kidney).

Timely revascularization reduces renal ischemia and renin–angiotensin–aldosterone system activation, reducing blood pressure and leading to clinical stabilization [8], finally, increasing survival and reducing further hospitalizations in these patients [16, 17]. A large study showed that in patients with RAS and history of recurrent heart failure, 82% of the successfully stented patients had no hospitalization during follow-up [13]. Patients with FPE due to RAS showed marked weight reduction following stenting, due to the intense diuresis [18].

Different studies showed long-term high mortality rates in RAS patients presenting with high-risk features, reaching 12% per year and up to 56% at four years [3, 19, 20]. In some studies, the survival benefit was observed only in patients with FPE [3] and, inconsistently between studies, in those with rapidly declining renal function [3]. In our group of high-risk RAS patients undergoing stenting, 33% mortality was observed on a follow-up of roughly 2 years.

Regarding postprocedural renal function, in our group was observed a rather equivocal response to angioplasty. Overall, improvement of the preprocedural renal function was observed in half of the patients. The expected result was somewhat masked by CIN, which occurred in a third of patients, entirely reversible in the majority of patients, but a net positive effect of angioplasty became finally visible only in two of six patients with CIN. On the other hand, when compared to baseline renal function (before the index hospitalization decline), pre-discharge creatinine levels were steady.

Similarly, renal failure improvement after stenting was inconsistent across different studies [21], with no change [22, 23] or even worsening in a significant proportion of the patients [19, 20]. However, there are studies showing that stenting improves or at least stabilizes renal function, especially in RAS patients with a rapid decline of function in the year before intervention [24–26], usually in patients with bilateral or solitary kidney stenosis [24]. Non-responders to revascularization are generally patients with stable reduced renal function before intervention [2].

Different studies showed inconsistent results regarding the progression to ESRD [3, 8, 13, 19, 23]. Ritchie et al. showed 18% progression to ESRD at 3.8 years, without difference between stenting and medical approach, in patients with high-risk features [3]. In our small group of stented high-risk RAS patients, roughly a quarter ended-up on hemodialysis after two years of follow-up. The predictors of ESRD were diabetes and baseline renal failure, suggesting as cause the progression of underlying renal parenchymal disease rather than renal vascular disease. Diabetes is an established independent cause of ESRD due to microvascular complications [8]. There was no improvement after revascularization in patients with proteinuria > 1 g/day [8]. In order to estimate the benefit of revascularization in ischemic nephropathy, the relative contribution of RAS and intrinsic nephropathy to renal failure should be assessed by urinalysis (proteinuria), renal size (by imaging), renal resistive indexes, serum creatinine or estimated glomerular filtration rate. Pre-treatment eGFR of < 30 ml/min and severely increased albumin to creatinine ratio were each independent predictors of worse outcome [27].

Predictors of a positive response to stenting are deterioration of renal function after angiotensin converting enzyme inhibitors, rapid decline of renal function, kidney dimension > 8 cm, no signs of cortical or interstitial fibrosis [7], or preoperative resistive index up to 0.75 [28, 29].

Studies have shown that revascularization has little impact on hypertension control (1–20%) [30, 31]. However, our study demonstrated better hypertension control after two years of follow-up, but this could not be entirely attributed to revascularization.

### Study limitations

Retrospective nature of the research and the low number of patients are the main drawbacks of our study. The results are attributed to procedures performed during a long period, implying different renal angioplasty techniques and devices. Baseline data on renal disease reversibility, as proteinuria, resistivity index and kidney size were lacking for analysis. In addition, functional evaluation of the severity of RAS was not available; however, in general, we treated tight stenoses (> 70%), leaving little doubt on their significance. We did not have a medically treated controlled group, because in patients with high risk RAS there is no equipoise between the two possible treating strategies (stenting vs. conservative treatment), hence the descriptive nature of our study. As the follow-up was made by telephone contact, no direct clinical or laboratory data could be obtained.

## Conclusions

Percutaneous procedures are feasible and safe in patients with high-risk renal artery stenosis, especially in those with rapidly declining renal function, probably saving some of them from the immediate need for renal replacement therapy, but long-term results are negatively influenced by the precarious general and cardio-vascular status of these patients and by the pre-existing significant renal parenchymal disease, non-related to the renal artery stenosis.

## Data Availability

The datasets used and/or analyzed during the current study are available from the corresponding author on reasonable request.

## Abbreviations

RAS: Renal artery stenosis
FPE: Flash pulmonary edema
CIN: Contrast-induced nephropathy
ESRD: End-stage renal disease
eGFR: Estimated glomerular filtration rate
BP: Blood pressure
ACEi: Angiotensin converting enzyme inhibitor
ARB: Angiotensin receptor blocker

## Declarations

### Study design

Retrospective descriptive analysis.
