# Correction: Outcomes after stenting of renal artery stenosis in patients with high-risk clinical features

**DOI:** 10.1186/s43044-024-00445-x

**Published:** 2024-02-01

**Authors:** Calin Homorodean, Mihai Claudiu Ober, Mihail Spinu, Maria Olinic, Dan-Alexandru Tataru, Horea Laurentiu Onea, Alexandru Achim, Leontin Florin Lazar, Romana Homorodean, Balasz Deak, Dan Mircea Olinic

**Affiliations:** 1 Medical Clinic No. 1, “Iuliu Hatieganu” University of Medicine and Pharmacy, Cluj-Napoca, Cluj Romania; 2 Cluj County Emergency Clinical Hospital, 3-5, Clinicilor Street, 400006 Cluj-Napoca, Cluj Romania

**Correction: The Egyptian Heart Journal (2024) 76:4** 10.1186/s43044-024-00435-z

Following publication of the original article [1], the authors identified an error in the author name of Horea Laurentiu Onea.

The incorrect author name is: Horea Lauretiu Onea.

The correct author name is: Horea Laurentiu Onea.

The author group has been updated above and the original article [1] has been corrected.

## References

[1] Homorodean C, Ober MC, Spinu M (2024). Outcomes after stenting of renal artery stenosis in patients with high-risk clinical features. Egypt Heart J.

